# Sarcoplasmic reticular Ca^2+^-ATPase inhibition paradoxically upregulates murine skeletal muscle Na_v_1.4 function

**DOI:** 10.1038/s41598-021-82493-w

**Published:** 2021-02-02

**Authors:** Sean X. Liu, Hugh R. Matthews, Christopher L.-H. Huang

**Affiliations:** 1 Physiological Laboratory, University of Cambridge, Cambridge, CB2 3EG UK; 2 Department of Biochemistry, University of Cambridge, Cambridge, CB2 1QW UK

**Keywords:** Biological techniques, Biophysics, Physiology

## Abstract

Skeletal muscle Na^+^ channels possess Ca^2+^- and calmodulin-binding sites implicated in Nav1.4 current (*I*_Na_) downregulation following ryanodine receptor (RyR1) activation produced by exchange protein directly activated by cyclic AMP or caffeine challenge, effects abrogated by the RyR1-antagonist dantrolene which itself increased *I*_Na_. These findings were attributed to actions of consequently altered cytosolic Ca^2+^, [Ca^2+^]_i_, on Na_v_1.4. We extend the latter hypothesis employing cyclopiazonic acid (CPA) challenge, which similarly increases [Ca^2+^]_i_, but through contrastingly inhibiting sarcoplasmic reticular (SR) Ca^2+^-ATPase. Loose patch clamping determined Na^+^ current (*I*_Na_) families in intact native murine gastrocnemius skeletal myocytes, minimising artefactual [Ca^2+^]_i_ perturbations. A bespoke flow system permitted continuous *I*_Na_ comparisons through graded depolarizing steps in identical stable membrane patches before and following solution change. In contrast to the previous studies modifying RyR1 activity, and imposing control solution changes, CPA (0.1 and 1 µM) produced persistent increases in *I*_Na_ within 1–4 min of introduction. CPA pre-treatment additionally abrogated previously reported reductions in *I*_Na_ produced by 0.5 mM caffeine. Plots of peak current against voltage excursion demonstrated that 1 µM CPA increased maximum *I*_Na_ by ~ 30%. It only slightly decreased half-maximal activating voltages (*V*_0.5_) and steepness factors (*k*), by 2 mV and 0.7, in contrast to the *V*_0.5_ and *k* shifts reported with direct RyR1 modification. These paradoxical findings complement previously reported downregulatory effects on Nav1.4 of RyR1-agonist mediated *increases* in *bulk* cytosolic [Ca^2+^]. They implicate possible *local* tubule-sarcoplasmic triadic domains containing *reduced* [Ca^2+^]_TSR_ in the observed upregulation of Nav1.4 function following CPA-induced SR Ca^2+^ depletion.

## Introduction

The skeletal muscle Na^+^ channel Na_v_1.4 structure possesses several potential Ca^2+^ and calmodulin (CaM) binding or modulation sites. These may permit (a) Ca^2+^ binding to one or more C-terminal EF-like hand motifs^1^, (b) CaM binding to a C-terminal isoleucine-glutamine (IQ) domain following Ca^2+^ binding to EF-hand motifs on the CaM itself^2,3^, and (c) Ca^2+^/CaM-binding to a III–IV loop site^4^. It also possesses (d) sites phosphorylatable by CaM kinase II (CaMKII) itself regulated by Ca^2+^/CaM^5^ and (e) a phosphorylation site for phosphokinase C^6^. One or more of these sites may underly in vitro single-cell patch-clamp reports that elevating [Ca^2+^]_i_ to ~ 2 µM through rapid photo-release and Ca^2+^ overspill from neighbouring Ca^2+^ channels reduced peak Na^+^ current densities, *I*_Na_, in Na_v_1.4-transfected HEK293 cells and skeletal muscle cell lines, effects abrogated by intracellular BAPTA^7^. These effects were abolished with mutations in either the Ca^2+^-binding-EF hands on CaM or the Na_v_1.4 C-terminal IQ domain, implicating CaM binding to the IQ domain in the [Ca^2+^]_i_-mediated Na_v_1.4 modulation^2,7,8^.

These findings were extended to native intact working murine skeletal myocytes using loose patch clamp methods that avoided further artefactual intracellular Ca^2+^ perturbations arising from the intrapipette EGTA used in conventional patch clamping. Ryanodine receptor (RyR1) agonists known to gate sarcoplasmic reticular (SR) store Ca^2+^ release reversibly downregulated *I*_Na_. These effects of both the Epac activator 8-(4-chlorophenylthio)adenosine 3′,5′-cyclic monophosphate (8-CPT) and RyR1 agonist caffeine were abrogated by the RyR1 antagonist dantrolene^9,10^. Dantrolene itself contrastingly produced increases in *I*_Na_. Murine cardiac muscle showed concordant effects of enhanced SR Ca^2+^ release either with 8-CPT application^11^ or in RyR2-P2328S genetic variants^12^.

The present experiments test and extend the resulting hypothesis implicating alterations in [Ca^2+^]_i_ in such a feedback modification of Na_v_1.4 function suggested by the latter reports. They now explore effects of cyclopiazonic acid (CPA) challenge. In common with caffeine^13^ this increases bulk cytosolic [Ca^2+^]_i_^14^. However, rather than enhancing RyR-mediated Ca^2+^ release, it does so through inhibiting SR Ca^2+^-ATPase (SERCA). This reduces SR cytosolic Ca^2+^ uptake thereby depleting SR store Ca^2+^^15^. The experiments similarly employed loose patch clamping protocols, but these were further modified to permit pair-wise statistical comparisons of *I*_Na_ in the same established undisrupted patch before and following solution changes delivering the pharmacological challenge. This dealt with any between-patch *I*_Na_ variations reflecting previously reported non-uniform ion channel, including Nav1.4, distributions over the muscle fibre surface^16^.

We here report that in contrast to expectations from the previous experiments that modified RyR1-mediated Ca^2+^ release^9,10^, CPA paradoxically enhanced rather than reduced *I*_Na_. We reconcile these findings with those previous reports, in terms of a scheme in which at least some tubular (T) Nav1.4 are exposed to local T-SR triad Ca^2+^ concentrations [Ca^2+^]_TSR_ themselves influenced by terminal cisternal RyR1-mediated fluxes of SR Ca^2+^. The latter would be increased by the RyR1 agonists, caffeine and 8-CPT, but decreased by SR Ca^2+^ depletion produced by the SERCA inhibitor CPA. Such tubular Nav1.4 likely make a substantial contribution to measured *I*_Na_^17,18^, given that T-tubules comprise up to 80% of muscle fibre membrane surface area^19^.

## Results

### Inward loose patch currents obtained in response to depolarizing voltage steps

**Figure 1:**
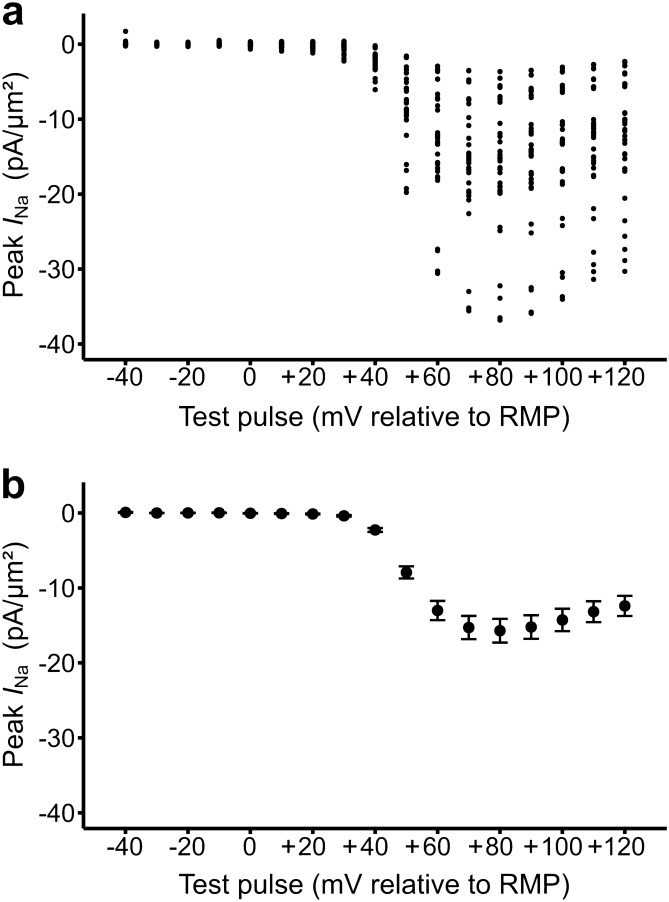
Current–voltage (*I–V*) relationships between patches. Data from (**a**) individual patches showing between-patch, normally distributed (n = 17; Shapiro–Wilkes test, P = 0.13), variations in peak currents obtained through the explored range of applied voltage steps, with (**b**) their means and standard errors.

*I*_Na_ currents elicited by the pulse protocol, normalized to patch areas giving current densities, initially rapidly increased to attain peak values before showing inactivation decays. This fulfilled expectations from previous studies similarly exploring *I*_Na_ using the loose patch clamp technique^9,10,16^. The peak currents obtained through the range of applied voltage steps were then quantified in current–voltage (*I–V*) relationships. Figure 1a demonstrates that these relationships assumed a similar form, but the current magnitudes varied between individual patches. Thus, whilst being normally distributed (Shapiro–Wilkes test, P = 0.13), the maximum peak currents (mean − 16.45 pA/µm^2^) showed significant variation (SD = 9.605, SEM = 2.329 pA/µm^2^; n = 17), confirming previous reports^16^. Nevertheless, the experimental design separated such between-patch variability from the observed changes associated with the pharmacological challenge under investigation.

### Control solution change protocols conserve patch and inward current characteristics

To fulfil the latter strategy, the experiments studied ionic currents before and following the solution changes introducing either control solution or solutions containing the test agents, making recordings before and following each solution change, from the same established patch. This permitted the pairwise comparisons of control and test results adopted here. This required maintenance of stable patches before and following the solution changes involving challenge with extracellularly applied cyclopiazonic acid (CPA) or otherwise. The externally applied CPA used here targeted cytosolic SERCA-mediated Ca^2+^ re-uptake into the SR, in contrast with the RyR1-specific agonist and antagonist agents employed in the previous studies. The CPA was used at 0.1/1.0 µM concentrations, well in excess of the reported 10–20 nM half-maximal concentration, to achieve maximal SERCA inhibition^15^. When recording from small cells, it is possible to effect rapid solution changes by stepping the interface between parallel continuous perfusing streams^20^. However this approach was not feasible in this case owing to the size of the tissue, so the entire bath solution was exchanged. The configuration used here nevertheless permitted relatively rapid solution changes by simultaneous removal of the initial whilst adding the new test solution, thereby maintaining bath solution level constant. This prevented vertical forces at the patch site due to changes in buoyancy forces acting on the muscle. This was despite reduction of the bath volume using the obturator insert to ensure completeness of the solution change.

**Figure 2:**
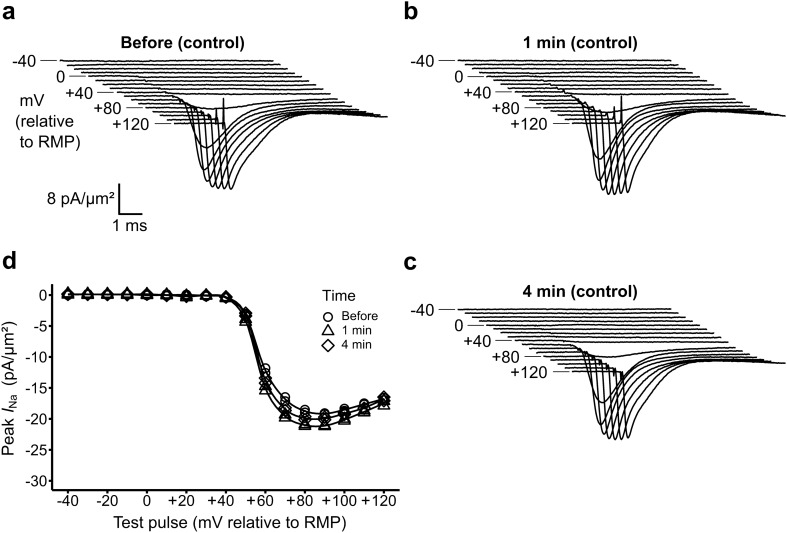
Currents and current–voltage (*I*–*V*) relationships through control solution changes. (**a**–**c**) Families of current traces obtained from a patch in response to voltage clamp steps applied relative to resting membrane potential (RMP), (**a**) before, (**b**) 1 min and (**c**) 4 min after solution change to K–H with identical composition to the original bath solution. Each trace shown is the average of two recordings obtained from the set of pulse protocols. It is displaced horizontally and vertically from the previous trace. Currents are shown over the cycle time range of 53–62 ms within the pulse protocol in which the voltage step was delivered at 55 ms. (**d**) Plots of peak current density against test pulse voltage excursion, comparing current–voltage (*I*–*V*) relationships between the same axes, illustrating a < 12% alteration in maximum peak current following the solution change.

Control experiments in which the solution changes re-introduced Krebs–Henseleit (K–H) solutions containing no CPA were performed to test the effectiveness and validity of this approach. These manoeuvres produced little change in patch seal resistances, which suggested the solution changes did not disrupt patch integrity. Currents obtained from the subsequent patch recordings before (Fig. 2a) and following the solution changes (Fig. 2b) were similar in form and magnitude, remaining so with the later recordings (Fig. 2c). Furthermore, currents obtained through protocols in which test voltage steps had amplitudes in increasing order closely matched those in which the test voltage steps were applied with amplitudes in decreasing order. This confirmed that patch and current characteristics were stable throughout the voltage clamp step protocol. They also yielded similar derived current voltage (*I*–*V*) relationships, with a stability confirmed in comparisons of results obtained before, and 1 min and 4 min following the solution change (Fig. 2d).

### Cyclopiazonic acid (CPA) challenge upregulates Na^+^ current

In the initial experiments testing the effects of the SERCA inhibitor CPA on the measured currents, the solution exchanger was first loaded with 50 ml of K–H solution containing 0.1 µM CPA and connected to the preparation. This concentration was previously reported to cause significant effects in Langendorff-perfused murine hearts^21^. Following establishment of the patch seal, a set of recordings using a bath solution containing no drug were then obtained. The solution change was then promptly performed. Further recordings of ionic current were then made in the presence of CPA starting at 1 min post-exposure.

**Figure 3:**
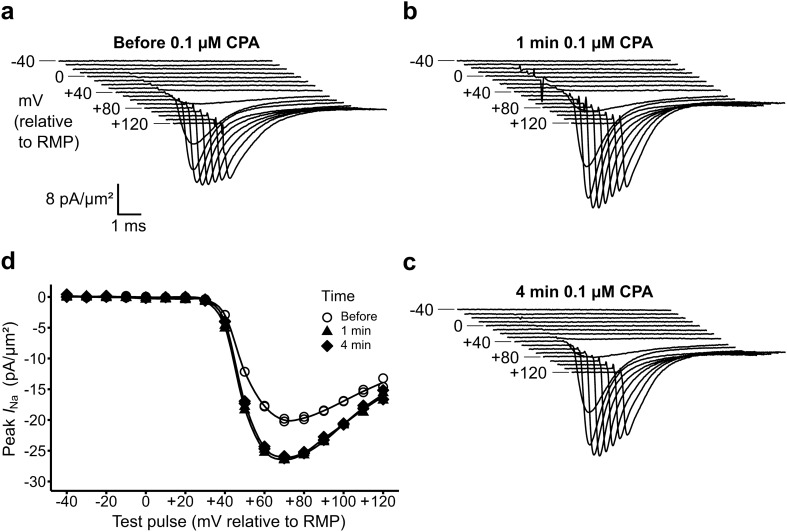
Effects of 0.1 µM CPA on currents and related *I*–*V* relationships. (**a**–**c**) Families of current traces obtained from applications of depolarizing voltage steps to a patch (**a**) before, (**b**) 1 min and (**c**) 4 min after solution change to K–H containing 0.1 µM CPA. (**d**) The resulting peak currents are plotted against test voltage excursions to give current voltage (*I–V*) relationships as in Fig. 2, here illustrating a ~ 20% alteration in maximum peak current following the solution change.

Figure 3 exemplifies ion current families obtained immediately before (a) and 1 min following (b) such a manoeuvre. Patch seal resistances remained stable through the solution change. In the presence of CPA, the peak current magnitudes were increased at all activating voltages by 1 min after the solution change. When obtaining the families of records, the currents were comparable through the pulse sequences applied using step amplitudes in increasing or in decreasing order. The current voltage (*I*–*V*) relationships showed increased inward current magnitudes but otherwise were similar in form. These effects stably persisted at 4 min following the solution change. However, alterations in patch characteristics, particularly the seal resistances, over longer, ~ 16 min, times prompted corroborative studies at higher, 1 µM CPA, concentrations than the 0.1 and 0.15 µM levels used previously in murine preparations^21^ as these might produce effects at earlier times. These employed less closely graded voltage clamp steps encompassing a smaller range of depolarizations. This permitted recordings to be completed in < 60 s whilst still permitting characterization of the current–voltage relationships derived below.

**Figure 4:**
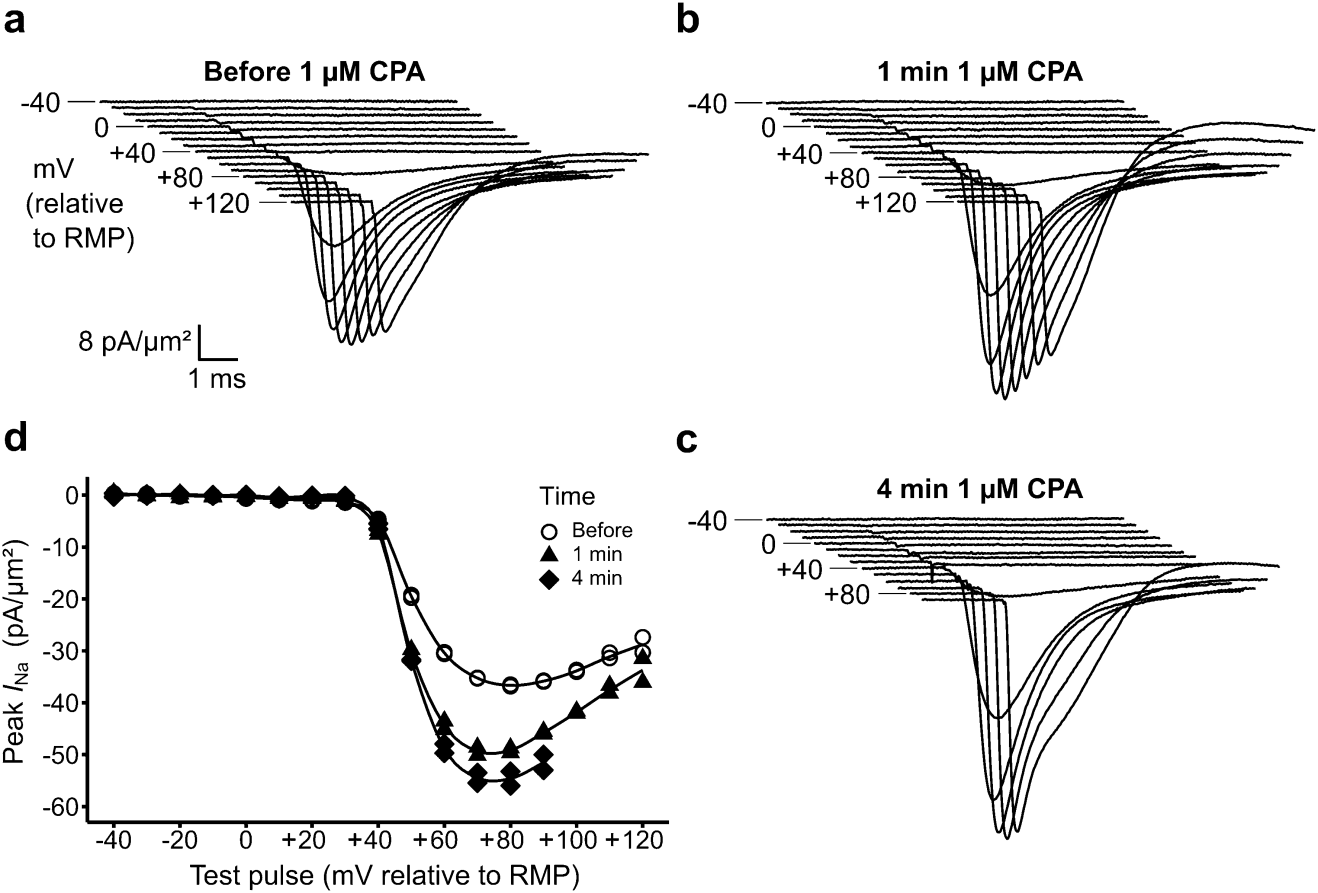
Effects of 1 µM CPA on currents and related *I–V* relationships. (**a**–**c**) Sets of current traces obtained from a patch (**a**) before, (**b**) 1 min and (**c**) 4 min after solution change to K–H solution containing 1 µM CPA. (**d**) Peak currents plotted against voltage excursion to give *I–V* relationships here illustrating a ~ 38% alteration in maximum peak current following the solution change.

These studies demonstrated similar findings at the CPA concentrations (1 µM) expected to supramaximally inhibit SERCA. They then gave more consistent, greater, increases in the currents observed at 1 min than did 0.1 µM CPA (Fig. 4a,b). Similarly altered current deflections were observed at the longer 4 min intervals (Fig. 4c). Furthermore, in all cases the initially observed current features at each voltage returned with the subsequently imposed voltage steps of decreasing amplitude. Current–voltage relationships obtained in the presence of 1 µM CPA resembled the relationships observed at the lower 0.1 µM concentration (Fig. 4d). These findings together implicate CPA in increasing the observed inward currents observed on loose patch clamping.

### CPA pretreatment abrogates effects of caffeine on I_Na_

**Figure 5:**
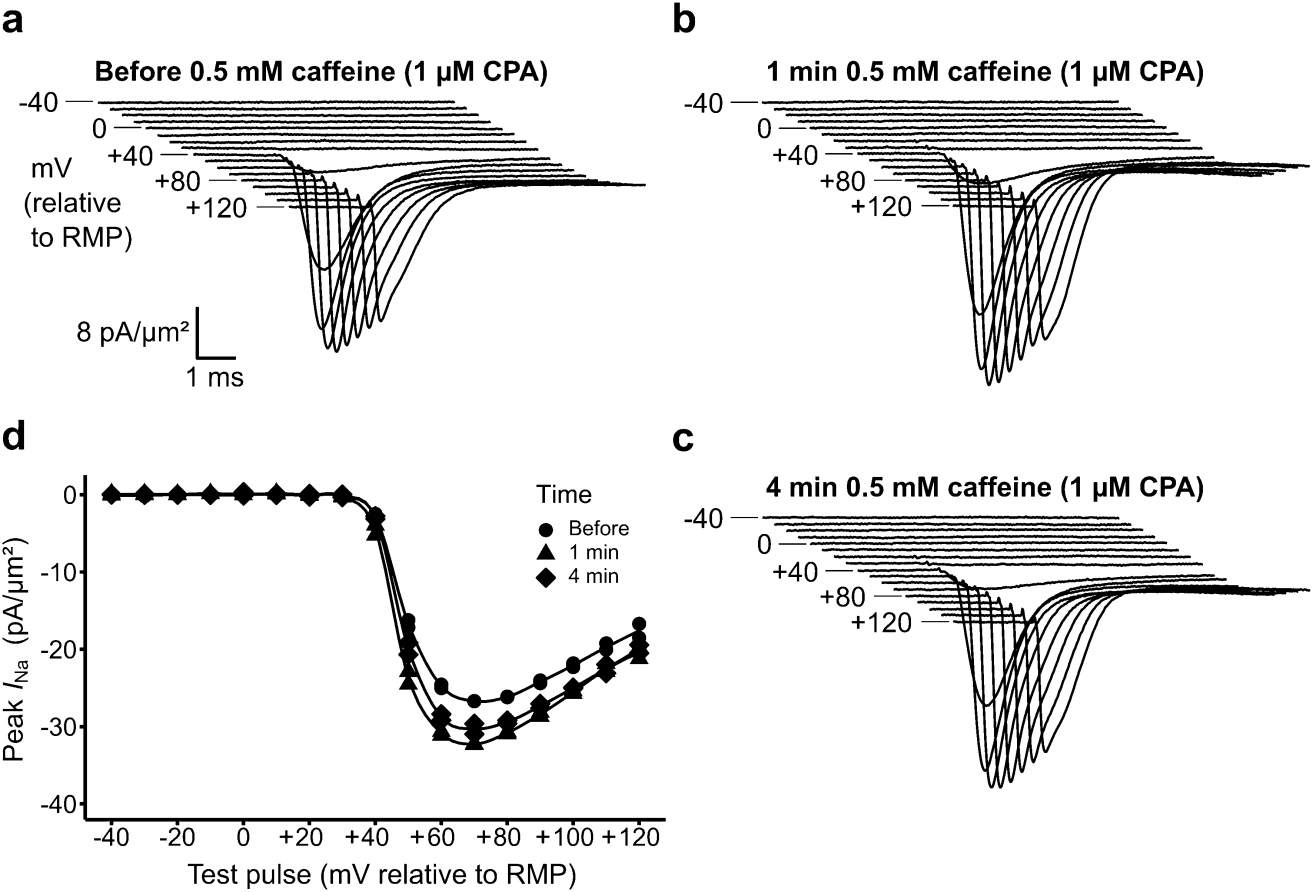
Effects of 0.5 mM caffeine challenge in the presence of 1 µM CPA. (**a**–**c**) Families of current traces from a patch (**a**) before, (**b**) 1 min and (**c**) 4 min after a preparation first pre-incubated with 1 µM CPA for 13 min underwent a solution change to one containing 0.5 mM caffeine in addition to 1 µM CPA. (**d**) Peak currents plotted against voltage excursion to give *I*–*V* relationships.

The present experiments using the SERCA inhibitor CPA, expected to deplete sarcoplasmic reticular (SR) Ca^2+^ stores, complemented previous experiments stimulating SR Ca^2+^ store release using the RyR1 agonist caffeine. Both agents were reported to increase bulk cytosolic [Ca^2+^]_i_^13,14^. However, in contrast to the sustained CPA action in increasing *I*_Na_ reported here, caffeine challenge caused decreases in *I*_Na_. But the latter effects were transient, reflecting its successive, RyR1-mediated, activation followed by inactivation of SR Ca^2+^ release^10^. Accordingly, further control experiments challenged CPA-treated muscle preparations with a superimposed addition of 0.5 mM caffeine (Fig. 5).

After setting up a preparation in K–H solution with no drug, the solution exchanger introduced 1 µM CPA. Patches were then obtained and a family of Na^+^ currents were recorded. Prior to this, the solution exchanger was loaded in readiness with 50 ml of K–H solution containing 1 µM CPA in combination with 0.5 mM caffeine. This permitted the prompt second solution change which thereby additionally introduced caffeine after ~ 10 min of pre-incubation in CPA. Further recordings were then taken from the same patch at 1 and 4 min as before. This challenge with multiple agents targeting Ca^2+^ homeostasis did not affect patch seal resistances, and recordings of families of Na^+^ currents remained possible. Furthermore, the findings contrasted with the previous reports in which 0.5 mM caffeine alone markedly decreased Na^+^ currents. Thus, this additional pre-exposure to CPA if anything, slightly increased the Na^+^ currents, a change in the opposite direction as that reported when caffeine challenge was applied following SR Ca^2+^ depletion^10^.

### Statistical quantification of current–voltage relationships

**Figure 6:**
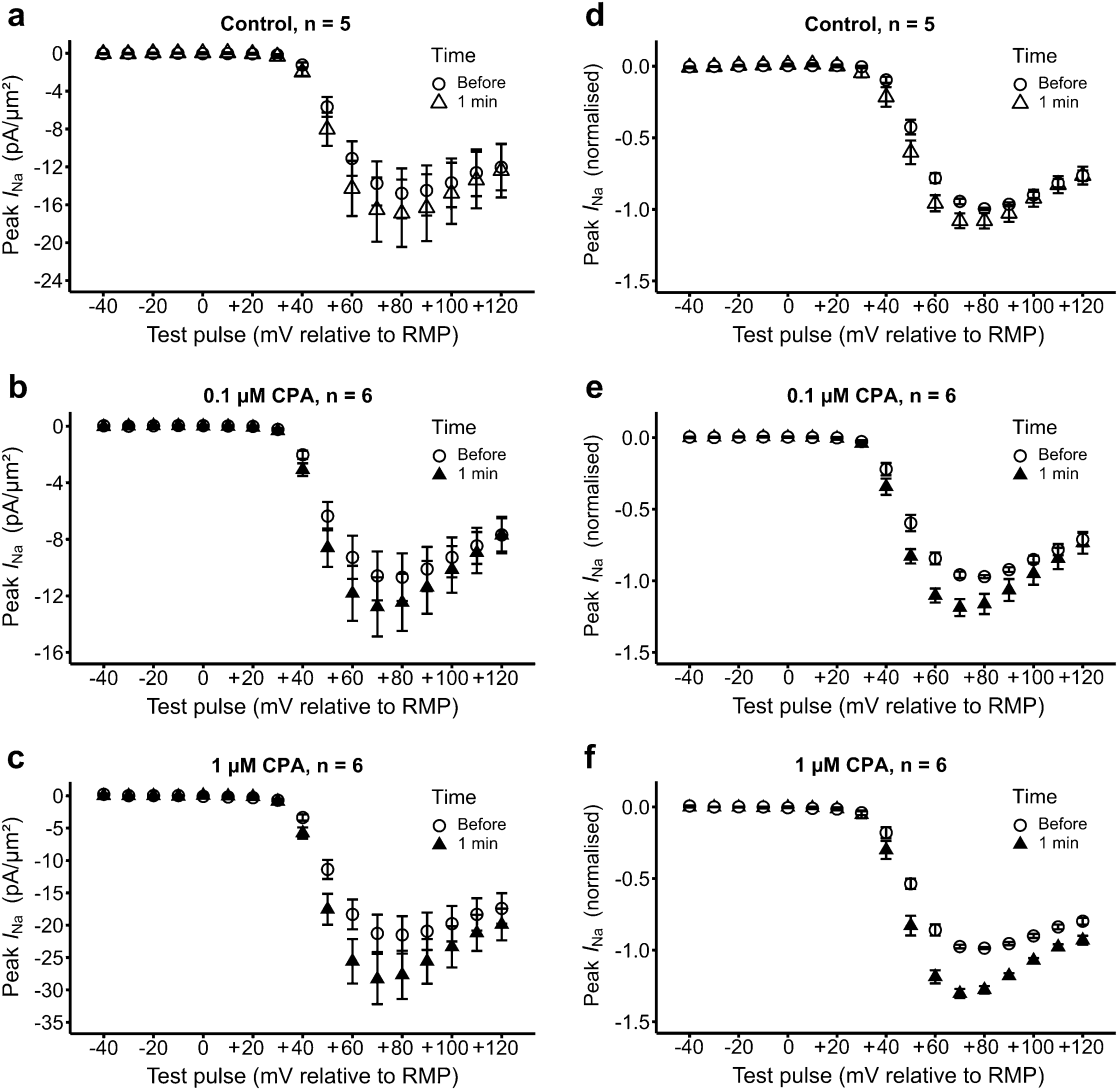
Comparison of current–voltage relationships through experimental manoeuvres. Mean (± SEM) peak currents from all patches plotted against voltage excursion giving *I–V* relationships, comparing measurements before and 1 min after solution change to one containing (**a**,**d**) 0 µM, (**b**,**e**) 0.1 µM CPA and (**c**,**f**) 1 µM CPA. Left panels (**a**–**c**) show comparisons of mean values of the observed currents with error bars representing standard errors of the mean (SEM) obtained from between-patch, unpaired, comparisons. Right panels (**d**–**f**) show *I–V* relationships normalized to maximum peak currents for each individual patch prior to CPA challenge thereby correcting for between-patch variations in peak current, representing their means and SEMs. Numbers of different patches (*n* value) are indicated above each graph; each patch contributed two measurements at a given voltage as part of sequences imposing progressively increasing and decreasing voltage steps.

Figure 6 summarizes the current–voltage relationships shown by the peak Na^+^ currents and their further analysis. It compares findings obtained prior to solution change with those obtained 1 min following alterations to solutions containing 0.0 µM (a, d; control), 0.1 µM (b, e) and 1 µM CPA (c, f) respectively. The resulting pairs of current–voltage (*I*–*V*) relationships remained consistent in shape, suggesting comparable activation characteristics. The left plots (a–c) show unpaired data showing peak currents from different patches plotted as means and standard errors of the mean (SEM) representing between-patch variations from the replicate experiments in the groups above. The right plots (d–f) illustrate the corresponding data normalized to the corresponding maximum peak current for each individual patch prior to CPA challenge and their SEMs. This analysis separates the between-patch variations in peak currents illustrated in Fig. 1 and reported on previous occasions^16^ from the paired within-patch alterations in Na^+^ current resulting from CPA challenge under examination.

As in previous reports, their rising phases for each patch were described by fitting them to a two-state Boltzmann function. Data within a range of voltages from − 40 mV relative to RMP to voltage excursions that produced the greatest magnitude of peak current (typically + 70 mV to + 90 mV relative to RMP) were fitted using a scaled Levenberg–Marquardt algorithm to the equation:

$$I={I}_{\mathrm{max}}-\frac{{I}_{\mathrm{max}}}{1+{\mathrm{e}}^{(V-{V}_{50}/k)}}.$$

Peak current densities (*I*, in pA/µm^2^) were thus related to clamped voltage excursion (*V*) through the maximum current (*I*_max_, in pA/µm^2^), voltage excursion at half-maximum current (*V*_50_, in mV) and a steepness factor (*k*, in mV). As recordings were taken before and after solution change from a single patch in each case, the results were paired and thus analysed by paired Student’s t-tests. Values of the mean, SEM and p values for each parameter are compared in Table 1.

**Table 1:** Results of fits of two-state Boltzmann equation before and immediately after solution change.

Condition	Variable	Before (mean ± SEM)	1 min (mean ± SEM)	*n* value	p value
Control	*I*_max_	− 14.86 ± 3.95	− 16.93 ± 5.24	5	0.2656
	*V*_50_	52.09 ± 1.74	48.33 ± 2.60	5	0.1174
	*k*	5.53 ± 0.41	5.15 ± 0.36	5	0.4471
0.1 µM CPA	*I*_max_	− 10.89 ± 2.46	− 12.82 ± 3.08	6	0.1375
	*V*_50_	48.21 ± 2.35	45.01 ± 1.90	6	0.0445*
	*k*	5.85 ± 0.65	4.90 ± 0.35	6	0.0476*
1 µM CPA	*I*_max_	− 21.74 ± 4.27	− 28.57 ± 5.67	6	0.0062*
	*V*_50_	49.14 ± 1.66	47.16 ± 1.71	6	0.0002*
	*k*	6.02 ± 0.39	5.30 ± 0.27	6	0.0476*

Three conditions shown, giving mean ± SEM values for between-patch maximum current, *I*_max_ (pA/µm^2^), half maximal voltage, *V*_50_ (mV) and steepness factor, *k* (mV). Significance of paired differences between results obtained before and 1 min following solution change expressed as the p value obtained from within-patch two-tailed paired Student’s t test.

*Denotes a significant difference where p < 0.05.

Solution changes in the control experiments introducing 0 µM CPA did not result in significant changes in *I*_max_, *V*_50_ or *k.* Although statistical comparisons did not suggest significant changes in *I*_max_ between control (0 µM) and 0.1 µM CPA concentrations, 1 µM CPA produced noticeable, statistically significant ~ 30% increases in *I*_max_. In contrast, both 0.1 µM and 1 µM CPA caused only small decreases (2–3 mV, < 1.0) in *V*_50_ and *k*, similar to those produced by the control experiments. These changes thus reflected the comparisons in Fig. 6 suggesting *I*–*V* relationships of similar form differing principally in their maximum currents.

## Discussion

Possible feedback, [Ca^2+^]_i_-mediated, in vitro, *I*_Na_ downregulation was first demonstrated in skeletal muscle cell lines and Na_v_1.4-transfected cultured HEK293 cells following rapid photo-release and Ca^2+^ overspill from neighbouring Ca^2+^ channels. This were abolished by mutations in the Ca^2+^-binding-EF hands on CaM or the Na_v_1.4 C-terminal IQ domain implicating direct or indirect Ca^2+^ binding to C-terminal functional groups in Nav1.4 ^1–3,7,8^. Subsequent experiments in intact loose patch clamped native murine muscle fibres correspondingly showed reduced or increased *I*_Na_ following respective activation or inhibition of SR Ca^2+^ release by direct pharmacological, caffeine and 8-CPT-mediated, RyR1 activation, or dantrolene-mediated RyR1 inhibition. Dantrolene pretreatment additionally abrogated the *I*_Na_ downregulatory effects of both 8-CPT and caffeine^9,10^.

The present studies further test and extend the resulting *I*_Na_ downregulation hypothesis. Previous fluo-3-AM and fura-PE3-AM Ca^2+^ fluorescence studies in rat soleus and oesophageal striated muscle reported that in common with caffeine, cyclopiazonic acid (CPA)^15,22^ increases bulk cytosolic Ca^2+^ concentrations, [Ca^2+^]_i_^13,14^. However, CPA acts by inhibiting sarcoplasmic reticular (SR) Ca^2+^-ATPase (SERCA)-mediated cytosolic Ca^2+^ re-uptake^15,22^ rather than activating RyR-mediated SR Ca^2+^ release. This additionally causes SR Ca^2+^ store depletion that itself can trigger store-operated Ca^2+^ entry (SOCE) potentially further increasing [Ca^2+^]_i_^23^. In common with the recent studies, experiments were performed on intact native skeletal myocytes. Use of the loose patch clamp technique avoided intracellular perturbations otherwise arising from conventional patch clamp access. Further introduction of novel solution change procedures permitted paired comparisons between families of *I*_Na_ elicited by progressively depolarizing test steps before and following applied solution changes within the same patch, without perturbing the patch seal. These matched comparisons avoided problems arising from previously reported between-patch *I*_Na_ variabilities reflecting non-uniform membrane ion channel distributions in muscle fibres^16^.

The *I*_Na_ recordings were unchanged by solution substitutions when these omitted CPA. However, in contrast to the previous observations in experiments using RyR1 activators^9,10^, substituting control for CPA-containing test solutions increased rather than decreased *I*_Na_. Thus, CPA (1 µM) statistically significantly increased mean maximum currents (*I*_max_) by ~ 31.4%. These effects were demonstrable from 1 min, and stably persisted in patches maintained for at least 4 min, following the solution change. The currents otherwise showed similar time courses, and similar steady-state half-maximum voltages, *V*_0.5_, and steepness factors, *k*.

These findings directly contrast with previously reported 81% decreases in *I*_max_, accompanied by shifts in *V*_0.5_, through similar, 1–10 min intervals following 0.5 mM caffeine challenge, despite the common effects of caffeine and CPA in increasing bulk cytosolic [Ca^2+^]_i_. Furthermore, whereas 0.5 mM caffeine had been reported to reduce *I*_Na_ when applied by itself^10^, we additionally report that CPA pre-treatment abrogated this effect. This parallels previous reports that CPA pre-incubation abrogated the arrhythmogenic effects of caffeine in murine hearts^21^. Both these effects of CPA thus more closely resembled that of dantrolene, despite its effects in decreasing rather than increasing [Ca^2+^]_i_. Dantrolene applied alone had caused significant, 14.9%, increases in *I*_max_^10^.

**Figure 7:**
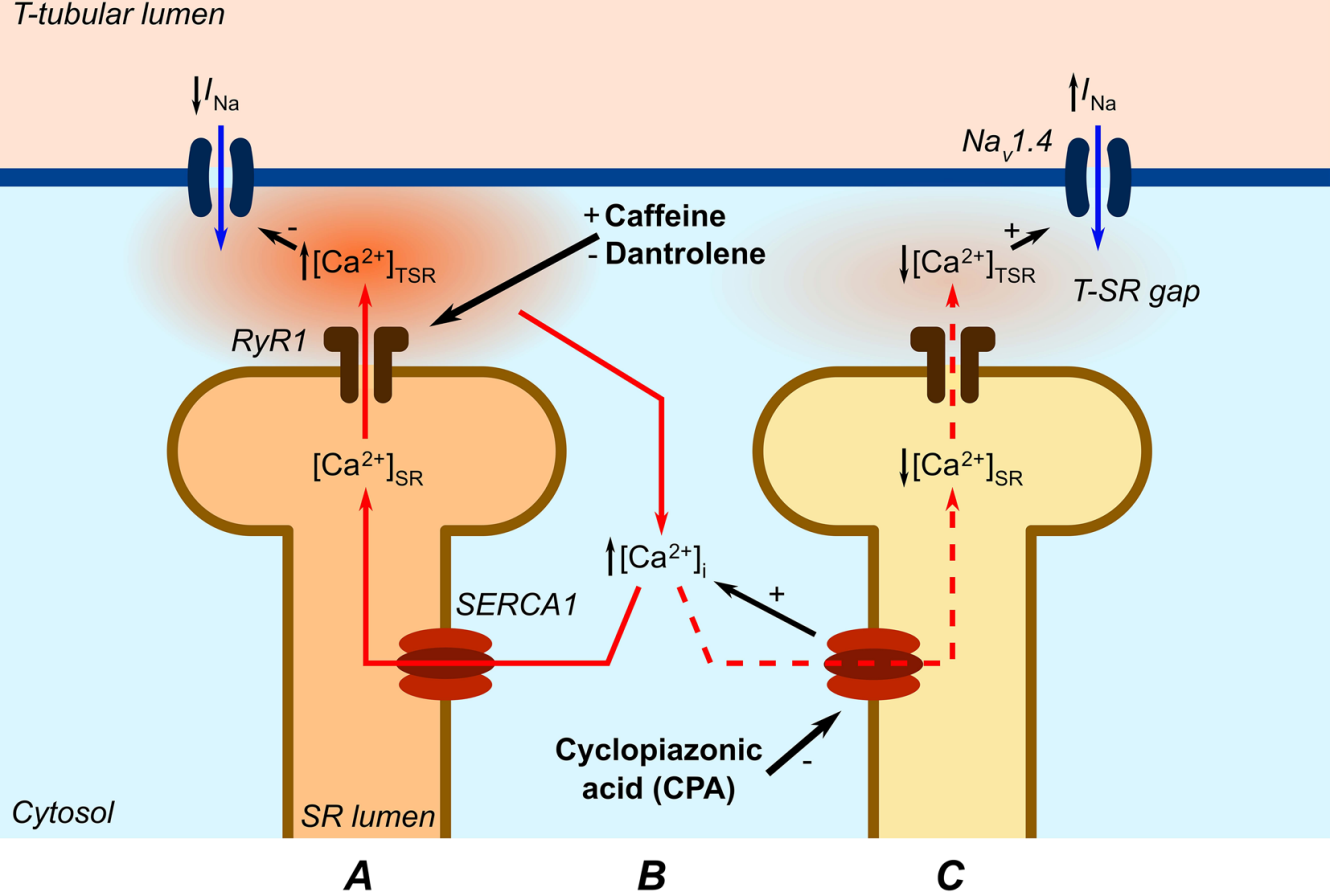
T-SR Ca^2+^ microdomain effects reconcile present with previous results. (**A**) In previous experiments^9,10^, caffeine and 8-CPT-induced RyR1-Ca^2+^ release channel opening *increased* fluxes of SR store Ca^2+^ into the T-SR space *increasing* its Ca^2+^ concentration, [Ca^2+^]_TSR_^13^, and ultimately (**B**) that of remaining cytosol, [Ca^2+^]_i_. This *inhibits I*_Na_. Such actions were antagonized by dantrolene. [Ca^2+^]_i_ is here also increased by (**B**) inhibiting cytosolic Ca^2+^ re-uptake by the SERCA inhibitor CPA^14^, but (**C**) the consequent depletion of SR Ca^2+^_,_ *reduces* SR store Ca^2+^ fluxes into the T-SR space. The latter correspondingly *reduces* [Ca^2+^]_TSR_ relieving its background inhibition of, thereby *increasing*, *I*_Na_.

The contrasting actions of RyR1 agonists and SERCA antagonists on *I*_Na_ implicate correspondingly contrasting effects on the local Ca^2+^ levels to which Nav1.4 is exposed, despite their similar reported effects on bulk cytosolic [Ca^2+^]_i_^13,14^. Besides the surface sarcolemma, Nav1.4 occurs in the transverse (T-) tubules^17,18^ themselves accounting for ~ 80% of total muscle membrane^19^, thereby contributing substantial *I*_Na_. T-tubular membranes come into close proximity to SR terminal cisternal membranes containing the RyR1-Ca^2+^ release channels, leaving electron-microscopically demonstrable narrow, ~ 15 nm, T-SR gaps permitting the Ca_v_1.1-RyR1 interactions strategic to excitation–contraction coupling^24–26^. Although ultimately continuous with remaining cytosol, T-SR gaps form restricted intracellular diffusion spaces close to the RyR1-Ca^2+^ release sites potentially generating microdomains with local Ca^2+^ concentrations, [Ca^2+^]_TSR_, distinct from the remaining bulk [Ca^2+^]_i_, that might modulate T-tubular, even if not surface membrane Na_v_1.4 function (Fig. 7).

The paradoxically contrasting results produced by RyR1 agonists, antagonists^9,10^, and SERCA inhibitors on *I*_Na_ may then reflect similarly contrasting changes in [Ca^2+^]_TSR_. Direct caffeine or 8-CPT induced background SR RyR1-Ca^2+^ channel activation would increase Ca^2+^ release into the T-SR gap increasing [Ca^2+^]_TSR_ thereby inhibiting T-tubular Na_v_1.4, as well as ultimately increasing bulk cytosolic [Ca^2+^]_i_, with dantrolene producing opposite or antagonistic effects^9,10^. CPA-mediated inhibition of SERCA-mediated Ca^2+^ uptake in the present experiments would similarly increase bulk cytosolic [Ca^2+^]_i_ but would deplete SR store Ca^2+^. The latter would instead reduce RyR1-mediated Ca^2+^ fluxes from SR lumen to T-SR space and diminish [Ca^2+^]_TSR_. This would explain the contrasting effects of CPA in increasing T-tubular Na_v_1.4 activity reported here. The depleted SR store Ca^2+^ would also explain the observed CPA-mediated abrogation of the known inhibitory actions of caffeine on *I*_Na._ Thus the SR Ca^2+^ depletion would compromise Ca^2+^ release into the T-SR gap through the activated RyR1-Ca^2+^ channels preventing any increase in [Ca^2+^]_TSR._ The present experiments thus complement possible future attempts at direct fluorescent Ca^2+^ indicator [Ca^2+^]_TSR_ measurements seeking such Ca^2+^ microdomains. However, the latter could be significantly hindered if not precluded by the small dimensions and dispersed nature of the T-SR compartment. Nevertheless, future theoretical diffusional modelling studies could well explore the extent to which released SR Ca^2+^ could accumulate within or become depleted from subcellular structures with architectures quantitatively matching those of T-SR junctions.

T-tubular as opposed to surface membrane Na_v_1.4 function might then possess distinct features reflecting the specific T-tubular roles in excitation–contraction coupling as opposed to surface membrane action potential propagation. These could be important in both normal and clinical neurological disorders of muscle function. Elevated [Ca^2+^]_TSR_ following sustained activity or in some human skeletal myopathies^27^ could inhibit tubular Na_v_1.4 function. Mutations in the Nav1.4 C-terminal EF hand-like domain possibly disrupting Ca^2+^-mediated inhibition of Nav1.4 function were recently implicated in a myotonic hyperexcitability disorder^28,29^. Finally, structural triad junction changes might also affect excitation–contraction coupling processes extending to *I*_Na_ regulation by Ca^2+^ signalling. For example, the T-SR gap is altered by normal physiological perturbations such as imposed exercise^30,31^. A mutation in junctophilin (JP2), thought involved in triad junction formation and maintenance was associated with increased myotube diameters and resting [Ca^2+^]_i_, and decreased RyR1-mediated Ca^2+^ release and excitation–contraction coupling gain^32^.

## Materials and methods

All experiments were regulated under the Animals (Scientific Procedures) Act 1986 Amendment Regulations 2012 following ethical review and approval by the University of Cambridge Animal Welfare and Ethical Review Body (AWERB). All procedures fell within the scope of Schedule 1 of the UK Animals (Scientific Procedures) Act (1986). The loose patches were made on single fibres within intact muscle preparations studied before and following challenge with extracellularly applied CPA directed at the intracellular SERCA target^15^. This involved solution change protocols permitting within-patch comparisons in view of established between-patch variations in maximum Na^+^ current^16^. This restricted experiments to each applying a single protocol to any one patch and studying one patch in a single fibre only within each dissected muscle.

### Tissue preparation

C57BL6 wild type mice were killed by cervical dislocation (Schedule I, UK Animals (Scientific Procedures) Act 1986) by Home Office licensed personnel and immediately dissected. All exposed tissues were bathed in Krebs–Henseleit (KH) extracellular solution (mM: 130 NaCl, 4.0 KCl, 1.2 HEPES, 1.0 MgCl_2_, 1.8 CaCl_2_, 10 glucose and 2.0 Na-pyruvate; pH adjusted to 7.4 using 1 M NaOH) throughout. The skin was reflected to expose gastrocnemius muscle. The overlying biceps femoris muscle was dissected from its superficial surface. The Achilles tendon was ligatured, bisected distally, and gastrocnemius and soleus muscles reflected. Their proximal attachments were freed by bisecting the femur, tibia and fibula, and all other muscles ~ 2 mm above and below the knee joint. The resulting preparation was immediately immersed in KH solution.

### Bath setup and solution exchanger

**Figure 8:**
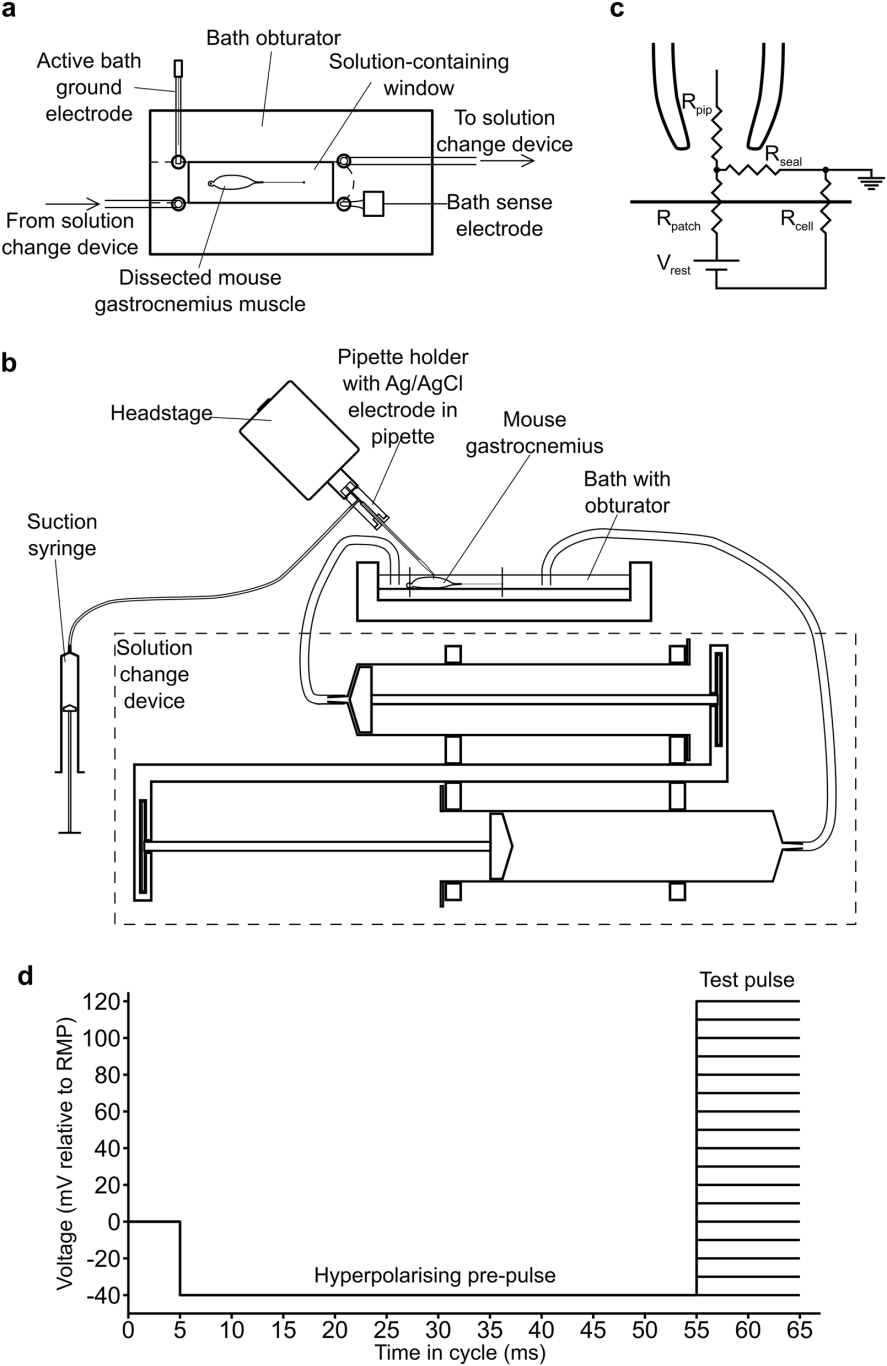
Experimental configuration. (**a**) Top view of bath setup for loose patch clamp. Scheme showing the bath obturator insert with pinned muscle preparation. Holes allow insertion of the bath sense and ground electrodes, used to actively clamp the bath potential, and tubes for solution exchange. They are continuous with the bath through cut-outs below the surface, demarcated by the dashed lines. (**b**) Side view of loose patch clamp setup showing micromanipulator-mounted headstage with recording pipette, holder, suction syringe and solution exchanger. (**c**) Equivalent circuit for loose patch clamp electrode on muscle membrane. The solution in the pipette, of resistance *R*_pip_, is clamped at pipette voltage, *V*_pip_. The voltage error arising from current flow through the patch, represented by patch resistance *R*_patch_ and the cell membrane resistance *R*_cell_, along with the pipette resistance (*R*_pip_) is corrected by bridge circuit in the loose patch clamp amplifier^9,10^. The cell is at its resting membrane potential, RMP, represented by the resting voltage *V*_rest_ during establishment of the patch. (**d**) Voltage clamp step protocol consisting of hyperpolarizing pre-pulse to (RMP − 40) mV followed by a variable test pulse starting at − 40 mV altered in 10 mV increments with successive pulse cycles to a test voltage of (RMP + 120) mV, followed by test steps in the reverse order from (RMP + 120) to (RMP − 40) mV.

The dissected muscle was placed into the adapted bath containing KH solution and fixed to its Sylgard surface using two A1 insect pins. One was inserted through the proximal bony structures, and the thread attached distally was tied around the other, holding the muscle under slight tension. A bespoke-fitting 3D-printed bath obturator was inserted to reduce bath volume from ~ 30 to 4 ml and hold the necessary tubes and electrodes (Fig. 8a). This together with a solution exchanger permitted rapid, complete solution changes minimally disturbing the patch. This used KH solution containing cyclopiazonic acid (CPA; Sigma-Aldrich, UK) introduced from a 10 mM stock in DMSO or containing vehicle alone. It employed two reciprocally connected syringes such that injection of a solution into the bath by one withdrew solution at the same rate by the other keeping bath fluid levels constant (Fig. 8b). The made-up solutions had been stored in small containers equilibrated for > 1 h prior to experimentation to a 20 °C temperature monitored by thermocouple. The 50 ml syringe capacities, far exceeding the obturated bath volume, ensured complete exchange in a process taking < 60 s. Their tubes were inserted into the bath obturator (Fig. 8a) as were the bath active ground current injection electrode (a stainless-steel wire) and the bath ground-sensing electrode (an ensheathed Ag/AgCl electrode containing KH solution) which actively maintained the bath at ground potential.

### Loose patch pipette manufacture and deployment

Borosilicate glass capillary tubes (GC150-10; Harvard Apparatus, Cambridge, UK) were pulled by a Flaming/Brown micropipette puller (Model P-97 Sutter Instrument Co., Novato, CA) using a 5-step program to achieve a progressive taper. The tips were visualized under a microscope with a calibrated eyepiece graticule at 250× magnification and scored using a diamond knife mounted perpendicularly to its long axis at a position giving a 30 µm internal diameter. A transverse force applied distal to this site produced a clean perpendicular fracture. The tips were fire-polished using an electrically heated nichrome wire under 400× magnification. The pipettes were bent through 45° at 1 mm from the tip, by heating with a nichrome filament and a transverse displacing force. Internal tip diameters were measured under 1000× magnification. Pipettes with 20–30 µm diameters were selected for use. A recording pipette was inserted into a pipette holder with a chloridized silver wire. This was connected to the headstage of the loose patch amplifier and held at 45° such that the pipette tip contacted the muscle surface perpendicularly (Fig. 8b). KH solution was drawn up from the bath using the suction syringe to provide electrical continuity through an Ag/AgCl electrode.

### Loose patch clamp recording

The custom-built loose patch amplifier circuit compensated for voltage drops across the pipette resistance in series with the patch and the current flow through the relatively low seal resistance of the loose patch configuration (typically < 2 MΩ compared to many GΩ in tight patch). Its variable resistances were adjusted to match the voltage drop across the pipette and seal resistances so that the membrane patch was clamped to the command potential and the circuit output corresponded to the current flowing through the patch only (Fig. 8c). Voltage clamp steps were delivered using an IBM-compatible computer. As the pipette tip being clamped was extracellular to the patch, the applied voltage steps produced membrane potential excursions of opposite sign to the conventionally expressed membrane potential. They took place relative to the resting membrane potential (RMP); they are accordingly referred to as such in this report.

The pulse protocols each began with a hyperpolarizing pre-pulse of − 40 mV relative to the resting membrane potential (RMP) to relieve any residual Na_v_1.4 inactivation at the RMP. This was then followed by a variable test pulse starting at (RMP − 40) mV and altered in + 10 mV increments with each successive cycle until a test voltage of (RMP + 120) mV was reached (Fig. 8d). The series of voltage steps was then repeated, but with the test voltage steps taking the opposite order running from (RMP + 120) to (RMP − 40) mV with 10 mV decrements.

Comparison of currents from these two sequences made it possible to check for stability of patch clamp current characteristics. A full set of pulses thus consisted of 34 cycles of such voltage steps, each of which were delivered at 2 s intervals. These permitted a derivation of current–voltage curves reflecting channel activation, an experimental approach that had been independently corroborated in inactivation protocols in previous studies employing the loose patch clamp technique^9,10^. Residual leak was corrected for by the P/4 protocol, whereby four voltage steps of opposite sign and a quarter in magnitude to the test pulse were delivered immediately after it. The control P/4 pulses traversed voltages that would not activate any voltage-gated conductances giving currents that represented leak currents for removal by summation with the test pulse record.

With the pipette tip immersed in the bath solution, the bath clamping was adjusted for offset so that the pipette electrode recorded zero voltage. The pipette resistance was compensated through its corresponding variable resistance, under guidance from an imposed square wave voltage clamp waveform viewed on an oscilloscope. The pipette tip was positioned over the centre of the convex surface of the gastrocnemius muscle, and slowly lowered using the fine vertical micromanipulator control under dissection microscope viewing. After achieving substantial cell surface contact producing a change in resistance at the pipette tip indicated by a deflection in the oscilloscope trace, gentle suction was applied, and the seal resistance compensated. A 15 ms depolarizing step of + 100 mV relative to RMP was applied to test patch viability. If the resulting current recording did not show a clear-cut Na^+^ current of suitable shape or magnitude, the patching procedure was re-attempted at an adjacent site. Viable patches were tested over a range of clamped voltage steps (Fig. 8d) to obtain a family of current responses. The solution change was carried out by sliding the connected plungers fully across in one continuous motion whilst observing the square wave on the oscilloscope. Small changes (< 5%) in seal resistance required readjustment of the corresponding resistance to cancel; large changes signalled disruption of the patch. The voltage clamp step protocol was then repeated at 1 min, 4 min and 8–10 min after the solution change, for as long as patch seal resistance permitted.

### Statistical analysis

The currents derived from the pulse protocol applied to each patch were normalized to the estimated patch area calculated using the loose patch pipette diameter to give current densities, and expressed as means ± SEM. Following Shapiro-Wilkes tests for normality, values of currents, *I* (pA/µm^2^), half maximal voltage, *V*_50_ (mV) and steepness factor, *k* (mV) were subject to within-patch paired t-test comparisons of values before and following CPA challenge.

### Study limitations

The present explorations thus developed a novel experimental configuration and simultaneously applied this to demonstrate for the first time within-patch alterations in Nav1.4 properties following hitherto untested pharmacological manipulations of [Ca^2+^]_i_. Its validation involved added confirmatory test step sequences to confirm recording stability. The added cycles of voltage steps potentially limited the testable range of applied pulse protocols owing to alterations in seal resistances with time following patch establishment. The latter were noticeable at later, 4 min, times at the higher CPA concentrations. However, these did not preclude acquisitions of *I*_Na_ activation curves sufficient for full quantification and comparisons of current–voltage relationships before and following CPA challenge.

### Ethical approval

This study was carried out in compliance with the ARRIVE guidelines. This research has been regulated under the Animals (Scientific Procedures) Act 1986 Amendment Regulations 2012 following ethical review and approval by the University of Cambridge Animal Welfare and Ethical Review Body (AWERB). All procedures were completed by Home Office-licensed personnel and fell within the scope of Schedule 1 of the UK Animals (Scientific Procedures) Act (1986).

## Data Availability

The data that support the findings of this study are available from the corresponding author upon reasonable request.
